
JOURNAL INFORMATION
==============================
NLM Title Abbreviation: Neth Heart J
Journal ID: Neth Heart J
NLM Journal ID: 101095458

Netherlands Heart Journal

ISSN: 1568-5888
EISSN: 1876-6250
Publisher: Springer

ARTICLE INFORMATION
==============================
PMCID: PMC5691821
DOI: 10.1007/s12471-017-1060-6
Article ID: 1060
Article version: 1
Subjects: Thanks to Reviewers

Thanks to reviewers

Electronic publication date: 2017 Nov 13
Print publication date: 2017 Dec
Volume: 25
Issue: 12
First page: 699
Last page: 700
Copyright: © The Author(s) 2017
License: Open Access. This article is distributed under the terms of the Creative Commons Attribution 4.0 International License (http://creativecommons.org/licenses/by/4.0/), which permits unrestricted use, distribution, and reproduction in any medium, provided you give appropriate credit to the original author(s) and the source, provide a link to the Creative Commons license, and indicate if changes were made.
License URL: https://creativecommons.org/licenses/by/4.0/

issue-copyright-statement: © The Author(s) 2017

==============================
Neth Heart J (2017) 25:699–700
https://doi.org/10.1007/s12471-017-1060-6

THANKS TO REVIEWERS

### Thanks to reviewers

Published online: 13 November 2017
© The Author(s) 2017 This article is available at SpringerLink with Open Access.

**The Editorial Team would like to extend our gratitude to all reviewers for their time and advice that helped us to assess and improve the papers that were submitted to us in 2017. The reviewers' critical and constructive feedback greatly helped to raise the journal's quality. We would like to thank Pieter Postema for most frequent involved in the review process in 2017.**

Wim Aanhaanen
Ahmet Adiyaman
Pierfrancesco Agostoni
Marco Alings
Cor Allaart
Sebastiaan Bekkers
Yuri Blaauw
Christa Boer
Kirsten Boerlage-van Dijk
Lucas Boersma
Maria Bonou
Jan-Willem Borleffs
Bas Boukens
Richard Braam
Frank Bracke
Marc Brouwer
Werner Budts
Cyril Camaro
Bimmer Claessen
Chantal Conrath
Michiel Coppens
Maarten-Jan Cramer
Harry Crijns
Joost Daemen
Kevin Damman
Giuseppe D'Ancona
Jacques de Bakker
Joris de Groot
Nicolaas De Jonge
Admir Dedic
Lucas Dekker
Thijs Delnoij
Biniyam Demissei
Dennis den Uijl
Corstiaan den Uil
Dirk Donker
Arif Elvan
Richard Folkeringa
Tjeerd Germans
Thomaz Gorter
M. Handoko
Magdalena Harakalova
Richard Hauer
Robin Heijmen
Frederik Helsen
José Henriques
Rik Hermanides
Alexander Hirsch
Hüseyin Ince
Luc Jordaens
Isabella Kardys
Suzanne Kats
Bas Kietselaer
Paul Knaapen
Kirsten Kortekaas
Adriaan Kraaijeveld
Boudewijn Krenning
Michael Kuiper
Andrew Landstrom
Eva Lanters
Geert Leenders
Tim Leiner
Miguel Lemmert
Erik Lipsic
Roberto Lorusso
Justin Luermans
Alexander Maass
Thomas Mast
Folkert Meijboom
Wouter Meijers
Esther Meindersma
Martijn Meuwissen
Hendrik Nathoe
Klaus Neef
Vincent Nijenhuis
Robin Nijveldt
Rutger Jan Nuis
Rohit Oemrawsingh
Marish Oerlemans
Ki Park
Els Pieper
Marco Post
Pieter Postema
Saman Rasoul
Tienush Rassaf
Jeroen Schaap
Jasper Selder
Anke Smits
David Sprenkeler
Pieter Stella

Bruno Stricker
T. Szili Torok
Tim ten Cate
Rachel ter Bekke
Arco Teske
Dominic Theuns
Robert Tieleman
Pim Tonino
Ype Tuininga
Victor Umans
Tim van de Hoef
Julio van de Swaluw
Maarten van den Berg
Kim van der heiden
Jeroen van der Heijden
Manon Van der Meer
Pepijn van der Voort
Arie van Dijk
Paul van Dijkman
Berry van Gelder
Ramon van Loon
Vincent van Ommen
Jurren van Opstal
Alexander Van Rosendael
Niels van Royen
Henrik Vase
Freek Verheugt
Kevin Vernooy
Marije Vis
Michiel Voskuil
Thierry Wildbergh
Arthur Wilde
FF Willems
Janneke Wittekoek
Joanna wykrzykowska
Sing-Chien Yap
Matthias Zink

**Open Access** This article is distributed under the terms of the Creative Commons Attribution 4.0 International License (http://creativecommons.org/licenses/by/4.0/), which permits unrestricted use, distribution, and reproduction in any medium, provided you give appropriate credit to the original author(s) and the source, provide a link to the Creative Commons license, and indicate if changes were made.
